# Simultaneous Validation of Seven Physical Activity Questionnaires Used in Japanese Cohorts for Estimating Energy Expenditure: A Doubly Labeled Water Study

**DOI:** 10.2188/jea.JE20170129

**Published:** 2018-10-05

**Authors:** Hiroyuki Sasai, Yoshio Nakata, Haruka Murakami, Ryoko Kawakami, Satoshi Nakae, Shigeho Tanaka, Kazuko Ishikawa-Takata, Yosuke Yamada, Motohiko Miyachi

**Affiliations:** 1 Graduate School of Arts and Sciences, The University of Tokyo, Tokyo, Japan; 2 Faculty of Medicine, University of Tsukuba, Ibaraki, Japan; 3 Department of Physical Activity Research, National Institute of Health and Nutrition, National Institutes of Biomedical Innovation, Health and Nutrition, Tokyo, Japan; 4 Faculty of Sport Sciences, Waseda University, Saitama, Japan; 5 Department of Nutrition and Metabolism, National Institute of Health and Nutrition, National Institutes of Biomedical Innovation, Health and Nutrition, Tokyo, Japan; 6 Department of Nutritional Epidemiology and Shokuiku, National Institute of Health and Nutrition, National Institutes of Biomedical Innovation, Health and Nutrition, Tokyo, Japan

**Keywords:** physical activity questionnaire, doubly labeled water method, activity energy expenditure

## Abstract

**Background:**

Physical activity questionnaires (PAQs) used in large-scale Japanese cohorts have rarely been simultaneously validated against the gold standard doubly labeled water (DLW) method. This study examined the validity of seven PAQs used in Japan for estimating energy expenditure against the DLW method.

**Methods:**

Twenty healthy Japanese adults (9 men; mean age, 32.4 [standard deviation {SD}, 9.4] years, mainly researchers and students) participated in this study. Fifteen-day daily total energy expenditure (TEE) and basal metabolic rate (BMR) were measured using the DLW method and a metabolic chamber, respectively. Activity energy expenditure (AEE) was calculated as TEE − BMR − 0.1 × TEE. Seven PAQs were self-administered to estimate TEE and AEE.

**Results:**

The mean measured values of TEE and AEE were 2,294 (SD, 318) kcal/day and 721 (SD, 161) kcal/day, respectively. All of the PAQs indicated moderate-to-strong correlations with the DLW method in TEE (rho = 0.57–0.84). Two PAQs (Japan Public Health Center Study [JPHC]-PAQ Short and JPHC-PAQ Long) showed significant equivalence in TEE and moderate intra-class correlation coefficients (ICC). None of the PAQs showed significantly equivalent AEE estimates, with differences ranging from −547 to 77 kcal/day. Correlations and ICCs in AEE were mostly weak or fair (rho = 0.02–0.54, and ICC = 0.00–0.44). Only JPHC-PAQ Short provided significant and fair agreement with the DLW method.

**Conclusions:**

TEE estimated by the PAQs showed moderate or strong correlations with the results of DLW. Two PAQs showed equivalent TEE and moderate agreement. None of the PAQs showed equivalent AEE estimation to the gold standard, with weak-to-fair correlations and agreements. Further studies with larger sample sizes are needed to confirm these findings.

## INTRODUCTION

Previous epidemiological studies have reported that daily energy expenditure is associated with various health outcomes.^1^ In these large-scale studies, energy expenditure, measured as total energy expenditure (TEE) and activity energy expenditure (AEE), were mainly assessed using physical activity questionnaires (PAQs). However, PAQs have a major disadvantage with regard to their limited validity against objective measures of physical activity in terms of energy. To overcome this challenge, some PAQs used in European and American studies were validated against the gold standard (ie, the doubly labeled water [DLW] method) under free-living conditions.^2^ In contrast, PAQs used in large-scale cohort studies in Japan have been validated mainly against activity records^3^ and have been rarely validated against the DLW method, with the exception of IPAQ and JALS-PAQ.^4^ To fill this gap, we simultaneously tested the validity of seven PAQs commonly used in Japan for estimating energy expenditure in comparison to the DLW method. To maximize internal validity, our study population was limited to young or middle-aged individuals relatively familiar with completing PAQs. TEE includes basal metabolic rate (BMR), diet-induced thermogenesis (DIT), and AEE, whereas AEE directly reflects energy expended due to any type of physical activity. Therefore, AEE would be useful for further investigation of dose-response relationships between physical activity and health outcomes.^5^ In contrast, TEE is important to determine the estimated energy requirement for a given population. The findings of the present study will facilitate better choice of PAQs and may help in the development of quality PAQs for future large-scale cohort studies.

## METHODS

### Participants

A total of 21 healthy Japanese adults aged 20–50 years were recruited from the Tokyo metropolitan area using flyers, e-mail distribution, and verbal outreach. After excluding one participant with incomplete data, 20 adults (9 men; mean age, 32.4 [standard deviation {SD}, 9.4] years; mean body mass index, 21.3 [SD, 1.8] kg/m^2^) provided data for the primary analysis. The participants consisted of 12 researchers and eight students. None of the participants were involved in the development of any PAQs (neither original nor Japanese versions) tested in this study. All protocols were reviewed and approved by the Ethics Review Board of the National Institute of Health and Nutrition, Japan. All participants gave written informed consent prior to initiation of the study.

### Procedures

Fifteen-day daily TEE and BMR were measured using the DLW method and a metabolic chamber, respectively. For DLW experiments, DLW dosing was conducted in the laboratory after collection of a baseline urine sample. Each participant collected his/her urine in airtight containers for 8 days under free-living conditions for 15 days. After 15 days, urine samples were recovered^6^ and urine analysis was performed using an isotope ratio mass spectrometer (SerCon 20-20; SerCon Ltd., Crewe, UK) according to the procedures described previously.^7^ Calculation of TEE was based on the A6 equation of Schoeller et al with Racette isotope dilution space.^8^^,^^9^ The dilution space ratio between ^2^H and ^18^O was 1.036 ± 0.011 (ie, 1.021–1.056), which passed the quality check for analysis.^10^ After the 15th day, the participants also self-administered the seven PAQs. For the metabolic chamber experiment, the participants stayed overnight in the metabolic chamber, and their BMR was measured in the supine position for 30 minutes from 07:30 after awakening at 07:00 and lying quietly for more than 15 minutes. The activity energy expenditure was then calculated as TEE − BMR − 0.1 × TEE, as an estimate of DIT.

### PAQ instruments

The inclusion criteria for PAQs were as follows: (1) used in large-scale cohorts (>10,000 participants) or nationwide representative surveys in Japan; (2) reported in research articles on associations of physical activity with health outcomes; (3) capable of estimating TEE and AEE. Based on these criteria, we chose five PAQs used in Japanese cohort studies.^3^^,^^4^^,^^11^^–^^13^ Two other globally accepted PAQs were also administered to facilitate international comparisons and potential study integration.^14^^,^^15^ The seven selected PAQs were the Japan Public Health Center-based Prospective Study-PAQ short form (JPHC-PAQ Short) and its long form (JPHC-PAQ Long),^3^ the Japan Arteriosclerosis Longitudinal Study-PAQ (JALS-PAQ),^4^ the National Integrated Project for Prospective Observation of Non-communicable Disease Trends in the Aged 2010-PAQ (NIPPON DATA-PAQ),^13^ the Jichi Medical School Cohort Study-PAQ (JMS-PAQ),^11^^,^^12^ the International Physical Activity Questionnaire short form (IPAQ Short),^14^ and the Global Physical Activity Questionnaire (GPAQ).^15^ The characteristics of these PAQs are summarized in Table 1.

**Table 1.** Characteristics of physical activity questionnaires used in this study

Name	Recall period	Number of items	Domains	Assigned METs	Reference
Japan Public Health Center-based Prospective Study-PAQ short form (JPHC-PAQ Short)	Usual	3	None	Heavy physical work or strenuous exercise: 4.5 METs Sedentary activity: 1.5 METs Walking and standing: 2.0 METs Others^*^: 1.5 METs ^*^24 hours - the sum of the above three activities	Fujii et al^3^

Japan Public Health Center-based Prospective Study-PAQ long form (JPHC-PAQ Long)	Past year	9	Occupational (includes commuting) Leisure time Sleeping	Occupational Heavy physical work or strenuous exercise: 4.5 METs Sedentary activity: 1.5 METs Walking and standing: 2.0 METs Leisure time Slow walking or strolling: 3.0 METs Brisk walking: 4.0 METs Light-to-moderate exercise: 4.0 METs Vigorous exercise: 4.5 METs Sleeping: 0.9 METs Others^*^: 1.5 METs ^*^24 hours - the sum of all the above activities	Fujii et al^3^

Japan Arteriosclerosis Longitudinal Study-PAQ (JALS-PAQ)	Usual	14	Occupational Transportation Household chores Leisure time Sleeping	Extracted from compendium of physical activities Need to have permission and send raw data to the JALS research team.	Ishikawa-Takata et al^4^

National Integrated Project for Prospective Observation of Non-communicable Disease And its Trends in the Aged 2010-PAQ (NIPPON DATA-PAQ)	Usual	6	None	Vigorous: 5.0 METs Moderate: 2.4 METs Light: 1.5 METs Watching TV and other sedentary behavior: 1.1 METs Sleep/lying down: 1.0 METs The sum of time spent in the above 6 activities is corresponded to a full 24 hours.	Kannel and Sorlie^12^

Jichi Medical School Cohort Study-PAQ (JMS-PAQ)	None	5	Occupational Non-occupational Sleep	Heavy: 5.0 METs Moderate: 2.5 METs Light: 1.5 METs Sedentary: 1.1 METs Sleep: 1.0 METs The sum of time spent in the above 5 activities corresponded to a full 24 hours.	Hayasaka et al^10^ Shibata et al^11^

International Physical Activity Questionnaire short form (IPAQ Short)	Past week	9	None	Vigorous: 8.0 METs Moderate: 4.0 METs Walking: 3.3 METs Each activity must last for ≥10 minutes.	Craig et al^13^

Global Physical Activity Questionnaire (GPAQ)	Past week	16	Occupational Transportation Leisure time	Vigorous: 8.0 METs Moderate: 4.0 METs Sedentary: 1.5 METs Each activity must last for ≥10 minutes.	Bull et al^14^

METs, metabolic equivalents; PAQ, physical activity questionnaire.

### Calculation of TEE and AEE by PAQs

All of the PAQs described in Table 1 provided total METs·h/day according to published articles or standard scoring protocols. TEE from the PAQs was calculated in two approaches with varying simplicity, which can cover various needs in research, clinical, and health promotion settings. The first is a simpler approach that uses participants’ body weight to estimate TEE and BMR, while the other approach incorporated a validated predictive equation for BMR. The former approach calculated TEE as total METs·h/day × body weight (kg). Regardless of the PAQ, BMR was calculated as 24 × body weight (kg) × 0.91, and diet-induced thermogenesis was estimated as TEE × 0.1. A factor of 0.91 was applied to correct for slight differences between BMR and resting metabolic rate.^16^^,^^17^ BMR is generally assessed in the supine position in the post-absorptive state (ie, after ≥12-hour fast), whereas resting metabolic rate is calculated in the sitting position a few hours after consuming a meal. Finally, AEE was computed by PAQs as TEE − estimated BMR − 0.1 × TEE. The latter approach first estimated BMR using Ganpule’s prediction equation,^18^ which was developed specifically for Japanese adults. The equation adopts age, gender, height, and body weight as predictors and suggests excellent applicability for large-scale studies in Japan.^19^ TEE was then estimated as mean METs·h/day × (estimated BMR/0.91).^16^^,^^17^ Finally, AEE was calculated as TEE − estimated BMR − 0.1 × TEE.

### Statistical analysis

All data handling and statistical analyses were performed using R (3.2.4 for Windows 64-bit; R Foundation for Statistical Computing, Vienna, Austria), and *P* < 0.05 was taken to indicate statistical significance.

To test the equivalency in TEE and AEE between the PAQs and the DLW method, equivalence testing with commonly-used two one-sided *t* tests was performed using the R package “equivalence”. Equivalence margins were set at ±10% based on previous similar studies.^20^^,^^21^ Root mean squared errors (rMSE) are presented to describe individual variability. To demonstrate the correlations and agreements, we calculated Spearman’s rank order correlations and intra-class correlation coefficients (ICCs) with 95% limits of agreement (LoA). In this study, rank correlation coefficients and ICCs were evaluated as weak (<0.20), fair (0.20–0.49), moderate (0.50–0.79), and strong (≥0.80).

## RESULTS

The average physical activity level (computed as measured TEE divided by measured BMR) was 1.73 (SD, 0.22) of the participants, which was classified as normal (class II) in the Dietary Reference Intake for Japanese 2015,^22^ and the range of physical activity level was 1.42–2.44. Mean TEE and AEE measured using the combination of the DLW method and metabolic chamber were 2,294 (SD, 318) kcal/day and 721 (SD, 161) kcal/day, respectively. Using a weight-based approach, among the seven PAQs, only JPHC Short provided a significantly equivalent TEE estimate (ie, within 10%) to TEE determined by the DLW method (Table 2). The rMSE ranged from 242 (JPHC Short) to 738 kcal/day (IPAQ Short). Correlation coefficients were all significant (*P* < 0.05 for all) and varied from 0.57 (NIPPON DATA-PAQ) to 0.80 (IPAQ Short and GPAQ). Among the seven PAQs, JPHC-PAQ Short and JPHC-PAQ Long showed moderate agreements in TEE with the DLW method. Based on the BMR equation approach, JPHC-PAQ Short and JPHC-PAQ Long provided significantly equivalent estimates to the measured TEE by the DLW method. The rMSE ranged from 240 (JPHC-PAQ Short) to 683 kcal/day (IPAQ Short). Correlation coefficients were moderate or strong and all were significant (*P* < 0.05 for all), ranging from 0.65 (JMS-PAQ) to 0.84 (JPHC-PAQ Short). Similar to the weight-based approach, JPHC-PAQ Short and JPHC-PAQ Long showed significant and moderate agreements in TEE with the DLW method. We performed gender-stratified analyses and found no notable differences in TEE. The JPHC-PAQ Short showed no significant bias of TEE (weight-based approach) against DLW in both men and women (−24 and −50 kcal/day, respectively; *P* > 0.05) as well as JPHC-PAQ Long in women (−179 kcal/day; *P* > 0.05). In contrast, the other five PAQs significantly underestimated TEE in both men and women (*P* < 0.05).

**Table 2.** Validity of seven physical activity questionnaires (PAQs) in estimating total energy expenditure (*n* = 20)

	Mean	SD	Diff.	95% CI		*P* for equivalence^a^	rMSE	Spearman’s rho	*P* for correlation	ICC	95% LoA	
Doubly labeled water	2,294	318										
Calculated based on body weight												
JPHC-PAQ Short	**2,256**	**374**	**−39**	**−153**	**76**	**<0.01**	242	**0.77**	**<0.01**	**0.75**	**0.48**	**0.89**
JPHC-PAQ Long	2,106	393	−188	−317	−60	0.26	327	**0.74**	**<0.01**	**0.63**	**0.19**	**0.84**
JALS-PAQ	1,964	349	−330	−439	−222	0.97	401	**0.76**	**<0.01**	0.51	−0.10	0.82
NIPPON DATA-PAQ	1,896	290	−398	−523	−273	0.99	476	**0.57**	**0.01**	0.33	−0.11	0.70
JMS-PAQ	1,625	302	−669	−795	−544	1.00	719	**0.65**	**<0.01**	0.19	−0.06	0.55
IPAQ Short	1,593	341	−701	−811	−591	1.00	738	**0.80**	**<0.01**	0.23	−0.05	0.62
GPAQ	1,605	330	−689	−791	−587	1.00	721	**0.80**	**<0.01**	0.24	−0.04	0.63
Calculated based on estimated BMR												
JPHC-PAQ Short	**2,334**	**395**	**40**	**−73**	**154**	**<0.01**	240	**0.84**	**<0.01**	**0.77**	**0.52**	**0.90**
JPHC-PAQ Long	**2,177**	**400**	**−117**	**−235**	**1**	**0.03**	273	**0.79**	**<0.01**	**0.73**	**0.42**	**0.88**
JALS-PAQ	2,029	349	−265	−358	−171	0.78	329	**0.81**	**<0.01**	0.63	−0.07	0.88
NIPPON DATA-PAQ	1,959	287	−335	−448	−221	0.97	410	**0.71**	**<0.01**	0.43	−0.11	0.77
JMS-PAQ	1,681	316	−613	−737	−489	1.00	666	**0.65**	**<0.01**	0.23	−0.07	0.61
IPAQ Short	1,648	352	−647	−752	−542	1.00	683	**0.82**	**<0.01**	0.27	−0.05	0.67
GPAQ	1,662	351	−632	−736	−528	1.00	668	**0.81**	**<0.01**	0.28	−0.05	0.68

BMR, basal metabolic rate; CI, confidence interval; Diff., difference; GPAQ, Global Physical Activity Questionnaire; ICC, intra-class correlation; IPAQ, International Physical Activity Questionnaire; JALS, Japan Arteriosclerosis Longitudinal Study; JMS, Jichi Medical School Cohort Study; JPHC, Japan Public Health Center-based Prospective Study; LoA, limits of agreement; NIPPON DATA, National Integrated Project for Prospective Observation of Non-communicable Disease And its Trends in the Aged; rMSE, root mean squared error; SD, standard deviation.

^a^Equivalence margin was set at ±10% of the measured TEE (229 kcal/day), Bold values indicate statistical significance (*P* < 0.05).

As expected, the validity of PAQs for estimating AEE was generally lower than that for TEE (Table 3). Regardless of the weight-based or BMR equation-based approach, none of the PAQs had estimates significantly equivalent to the AEE measured by the DLW method. Correlation coefficients ranged from 0.02 to 0.54 for the weight-based approach and from 0.12 to 0.54 for the BMR equation-based approach. In both approaches, JPHC-PAQ Short and JALS-PAQ provided significant fair-to-moderate correlations for AEE, and only JPHC-PAQ Short showed significant moderate agreement in AEE with the DLW method. The remainder of the PAQs showed weak agreements in AEE. JPHC-PAQ Short had no significant bias of AEE (weight-based approach) in both men and women (66 and 36 kcal/day, respectively; *P* > 0.05) as well as JPHC-PAQ Long (−91 and −81 kcal/day, respectively; *P* > 0.05). The remaining five PAQs significantly underestimated AEE in both men and women (*P* < 0.05).

**Table 3.** Validity of seven physical activity questionnaires (PAQs) in estimating activity energy expenditure (*n* = 20)

	Mean	SD	Diff.	95% CI		*P* for equivalence^a^	rMSE	Spearman’s rho	*P* for correlation	ICC	95% LoA	
Doubly labeled water	721	161										
Calculated based on body weight												
JPHC-PAQ Short	770	199	49	−40	139	0.30	193	0.44	0.05	**0.44**	**0.02**	**0.73**
JPHC-PAQ Long	636	223	−85	−197	26	0.60	248	0.22	0.35	0.23	−0.18	0.59
JALS-PAQ	508	173	−213	−303	−124	1.00	283	**0.54**	**0.02**	0.19	−0.11	0.53
NIPPON DATA-PAQ	447	186	−274	−385	−164	1.00	358	0.02	0.92	0.04	−0.13	0.30
JMS-PAQ	203	169	−518	−628	−408	1.00	567	0.16	0.50	0.00	−0.06	0.11
IPAQ Short	174	203	−547	−657	−436	1.00	593	0.31	0.19	0.03	−0.05	0.19
GPAQ	185	198	−536	−648	−424	1.00	585	0.23	0.33	0.02	−0.05	0.16
Calculated based on estimated BMR												
JPHC-PAQ Short	798	214	77	−15	170	0.55	208	**0.46**	**0.04**	**0.43**	**0.03**	**0.72**
JPHC-PAQ Long	657	227	−64	−175	47	0.44	240	0.29	0.21	0.27	−0.17	0.63
JALS-PAQ	524	172	−197	−284	−110	1.00	268	**0.54**	**0.02**	0.22	−0.11	0.56
NIPPON DATA-PAQ	461	188	−260	−370	−150	1.00	347	0.12	0.61	0.05	−0.14	0.32
JMS-PAQ	210	179	−511	−624	−397	1.00	563	0.22	0.35	0.00	−0.06	0.12
IPAQ Short	180	208	−541	−653	−429	1.00	589	0.33	0.15	0.03	−0.05	0.19
GPAQ	193	205	−528	−642	−413	1.00	579	0.22	0.34	0.02	−0.05	0.17

BMR, basal metabolic rate; CI, confidence interval; Diff., difference; GPAQ, Global Physical Activity Questionnaire; ICC, intra-class correlation; IPAQ, International Physical Activity Questionnaire; JALS, Japan Arteriosclerosis Longitudinal Study; JMS, Jichi Medical School Cohort Study; JPHC, Japan Public Health Center-based Prospective Study; LoA, limits of agreement; NIPPON DATA, National Integrated Project for Prospective Observation of Non-communicable Disease And its Trends in the Aged; rMSE, root mean squared error; SD, standard deviation.

^a^Equivalence margin was set at ±10% of the measured AEE (72 kcal/day), Bold values indicate statistical significance (*P* < 0.05).

## DISCUSSION

This study simultaneously tested the validity of seven PAQs used in Japan for estimating energy expenditure (ie, TEE and AEE) against the standard DLW method in a population that consisted mainly of researchers and students. The mean TEE of the participants was comparable to the average estimated energy requirement for Japanese adults.^22^ We found moderate-to-strong correlations in TEE with the DLW method. Two PAQs showed significantly equivalent TEE estimates and moderate agreements between the PAQs and DLW. None of the PAQs showed estimates that were significantly equivalent to the measured AEE. Two PAQs, JPHC-PAQ Short and JALS-PAQ, produced significant and fair-to-moderate correlations for AEE. Only JPHC-PAQ Short provided significant and fair agreement with the DLW method.

A few PAQs used in Japanese cohorts seem to have similar or even better validity in TEE than those reported in previous studies, which were mainly conducted in non-Asian countries.^2^ A systematic review summarizing comparative studies on PAQs and DLW reported unadjusted correlation coefficients (Pearson’s or Spearman’s depending on the study) of 0.15 to 0.63.^2^ The corresponding values for our study ranged from 0.57 to 0.80 for the weight-based approach and from 0.65 to 0.84 for the BMR equation-based approach. Our validation coefficients in AEE also appeared to be non-inferior to the values presented in the literature. The above-mentioned systematic review reported unadjusted correlation coefficients in AEE of 0.05 to 0.39 between PAQs and DLW,^2^ with the exclusion of one exceptionally strong correlation of 0.83.^23^ The corresponding values from our study ranged from 0.02 to 0.54 for the weight-based approach and from 0.12 to 0.54 for the BMR equation-based approach. Taken together, these results indicate that the PAQs used in Japanese cohorts have non-inferior validity to those reported in previous studies.

Interestingly, the validity found in our study using subjective measures of PAQs was not markedly inferior to that obtained by objective measurements, such as use of an accelerometer. Our research team reported simultaneous validity of 12 wearable devices, which included four research models and eight consumer-based models, in estimating free-living TEE against the DLW on the identical Japanese participants as in the present study.^24^ The study showed rank-order correlation coefficients of 0.80 to 0.88. A systematic review summarized accelerometer validation studies, which included a Japanese monitor, against the DLW method.^25^ The correlation coefficients reported in this review varied considerably from 0.18 to 0.91 for TEE. The corresponding values in our study ranged from 0.57 to 0.80 for the weight-based approach and from 0.65 to 0.84 for the BMR equation-based approach. These observations support the use of PAQs for estimating energy expenditure in large-scale cohort studies in Japan.

Although the correlations were generally acceptable, almost all PAQs tested provided lower estimates of both TEE and AEE compared to those obtained using the DLW method. This underestimation using PAQs can be modified by replacing assigned METs to questioning items with larger values. As shown in Table 1, both the NIPPON DATA-PAQ^13^ and the JMS-PAQ^11^^,^^12^ assigned 5.0 METs to heavy/vigorous activities and 2.4–2.5 METs to moderate activities. These assigned METs are lower than the globally accepted values of 6.0–8.0 METs for heavy/vigorous activities and 3.0–4.0 METs for moderate activity.^14^^,^^15^ Therefore, modification of assigned METs, preferably based on the reference standard of the DLW, may lead to improved ability of PAQs to estimate energy expenditure.

This study had several strengths. We adopted the DLW and metabolic chamber methods for measurement of TEE and BMR, respectively. These two methods are considered reference standards for energy expenditure. We also administered the PAQs used in representative large-scale Japanese cohorts. This allowed us to provide findings useful to a large proportion of epidemiologists in this field. In particular, estimating AEE as a standard estimate of activity level may be useful for integrating and harmonizing physical activity as an exposure variable, which will help to better examine dose-response relationships of physical activity with various health outcomes.

This study also had a few limitations. The study population consisted of researchers and students more familiar with completing PAQs compared to the general population. Our small sample size (*n* = 20) was another major limitation. These limitations may have led to selection bias, suggesting limited generalizability.

In summary, all of the PAQs had moderate-to-strong rank correlations in TEE compared to the standard DLW method. Two PAQs (the JPHC-PAQ Short and the JPHC-PAQ Long) showed significantly equivalent TEE estimates and fair agreements compared with the DLW method. However, none of the PAQs showed estimates significantly equivalent to the measured AEE. The JPHC-PAQ Short and the JALS-PAQ provided significant and moderate correlations for AEE. Further studies with larger sample sizes are needed to confirm these findings.
